# CHILDHOOD ADVERSITY IS ASSOCIATED WITH MENTAL HEALTH BUT NOT COGNITIVE DECLINE IN OLDER ADULTS

**DOI:** 10.1093/geroni/igad104.3221

**Published:** 2023-12-21

**Authors:** James Lian, Kim Kiely, Bridget Callaghan, Kaarin Anstey

**Affiliations:** University of New South Wales, Sydney, New South Wales, Australia; University of Wollongong, Sydney, New South Wales, Australia; University of California, Los Angeles, Los Angeles, California, United States; University of New South Wales, Sydney, New South Wales, Australia

## Abstract

**Objective:**

The aim of this study was to examine the association between childhood adversity and mental health and cognition in older adults.

**Methods:**

The sample included older Australian adults from the Personality and Total Health (PATH) Through Life Project (N = 2551). Childhood adversity was measured using a 17-item scale of domestic adversities (e.g., poverty, neglect, physical abuse, verbal abuse) and modelled using cumulative risk analysis (LCA). Mental health was measured using four validated depression and anxiety questionnaires and cognitive impairment was determined by a clinically validated algorithmic diagnostic criteria. The association between childhood adversity and late-life mental health was estimated using generalised additive models, and the association with cognition utilised multiple logistic regressions. Models were adjusted for gender, race, and education.

**Results:**

Generalised additive models indicated that a greater number of cumulative adversities were associated with poorer scores on all four mental health measures. No notable interactions between ACEs and gender were observed. In contrast, there was no association between childhood adversity and cognitive impairment in any of the tested logistic regression models. No gender differences were observed and no interactions with education or genotype were found.

**Conclusion:**

Consistent with prevailing literature, our study provides additional evidence for the enduring effects of domestic childhood adversity on anxiety and depression in older adulthood. Furthermore, the absence of associations between early adversity and cognitive impairment indicates that other factors might play a more prominent role in determining late-life cognitive outcomes.

